# Perspective: Wearable Internet of Medical Things for Remote Tracking of Symptoms, Prediction of Health Anomalies, Implementation of Preventative Measures, and Control of Virus Spread During the Era of COVID-19

**DOI:** 10.3389/frobt.2021.610653

**Published:** 2021-04-14

**Authors:** Sarmad Mehrdad, Yao Wang, S. Farokh Atashzar

**Affiliations:** ^1^Department of Electrical and Computer Engineering, New York University, New York, NY, United States; ^2^Department of Biomedical Engineering, New York University, New York, NY, United States; ^3^Department of Mechanical and Aerospace Engineering, New York University, New York, NY, United States

**Keywords:** COVID-19, IoMT, smart wearables, spread control, AI for health, smart connected health, telemedicine, symptom tracking

## Abstract

The COVID-19 pandemic has highly impacted the communities globally by reprioritizing the means through which various societal sectors operate. Among these sectors, healthcare providers and medical workers have been impacted prominently due to the massive increase in demand for medical services under unprecedented circumstances. Hence, any tool that can help the compliance with social guidelines for COVID-19 spread prevention will have a positive impact on managing and controlling the virus outbreak and reducing the excessive burden on the healthcare system. This perspective article disseminates the perspectives of the authors regarding the use of novel biosensors and intelligent algorithms embodied in wearable IoMT frameworks for tackling this issue. We discuss how with the use of smart IoMT wearables certain biomarkers can be tracked for detection of COVID-19 in exposed individuals. We enumerate several machine learning algorithms which can be used to process a wide range of collected biomarkers for detecting (a) multiple symptoms of SARS-CoV-2 infection and (b) the dynamical likelihood of contracting the virus through interpersonal interaction. Eventually, we enunciate how a systematic use of smart wearable IoMT devices in various social sectors can intelligently help controlling the spread of COVID-19 in communities as they enter the reopening phase. We explain how this framework can benefit individuals and their medical correspondents by introducing Systems for Symptom Decoding (SSD), and how the use of this technology can be generalized on a societal level for the control of spread by introducing Systems for Spread Tracing (SST).

## 1. Introduction

SARS-CoV-2, also known as COVID-19, is a novel coronavirus that initiated a pandemic outbreak in December 2019. Due to the high infection rate and relatively low mortality rate, as well as the long incubation period, COVID-19 spread through more than 19 countries by late-January 2020 (Adhikari et al., 2020; Tang et al., 2020; Zhai et al., 2020). The aggressive nature of the virus besides the limited knowledge has resulted in high pressure on the healthcare systems (Wang C. et al., 2020). As the initial waves of this virus is being passed in some countries (Leung et al., 2020), many nations and states are going through phases of reopening (Ainslie et al., 2020; Olagnier and Mogensen, 2020), which suggests that active monitoring of symptom development and spread should be conducted more robustly while preventive measures are implemented in a multifaceted manner to mitigate (if not possible to prevent) the following waves of the pandemic.

In the absence of widely-available vaccine for different variants of the virus, and approved treatment during any pandemic, the only available solution is to implement preventive measures to be taken in an attempt to mitigate the virus' damage as much as possible until a reliable cure is found (Le et al., 2020). As of the time of writing this paper, no approved cure for COVID-19 has been found, and the research for finding a solution to end this pandemic is still ongoing (Li X. et al., 2020) with some limited access to vaccines for initial variants of the virus. A wide range of tests has been introduced for diagnosing infected cases such as CT Scans (Li and Xia, 2020) and Polymerase Chain Reaction (PCR) (Li Y. et al., 2020; Long et al., 2020). Research centers are acquiring knowledge about the virus to understand the infection mechanism (Zheng et al., 2020), alarming early symptoms (Sun et al., 2020), silent symptoms (e.g., “happy hypoxemia Guo et al., 2020; Tobin et al., 2020”), and the virus' function in the body (Chen et al., 2020).

Any possible solution that can facilitate faster and more accurate preventative actions (Adhikari et al., 2020), means of diagnosis (Wynants et al., 2020; Zhai et al., 2020), development of predictive models (for identifying symptoms' progress) (Liu et al., 2020), tracing, and monitoring (Hellewell et al., 2020) is highly beneficial and essentially needed by several policymakers and stakeholders (Ransing et al., 2020). These activities concern hospitalized patients, out-patients, and those who have not been diagnosed.

This article provides the authors' perspectives about the functionality of smart wearable IoMT technologies for early diagnosis of COVID-19 symptoms (including silent symptoms) at the individual level and for tracking the interpersonal interaction using which the spread of the virus within the society can be modeled. We argue that the same technology can be used beyond COVID-19 and for detection and tracking of any infectious disease which results in respiratory symptoms. We will discuss the existing techniques and technologies and will explain the existing technical challenges to be addressed. We explain the functionality of state-of-the-art biosensors and machine intelligence which can be fused in the context of wearable IoMT technology to address several “unmet needs.”

In this paper we categorize IoMT technologies as (a) Systems for Symptom Decoding (SSD), and (b) Systems for Spread Tracing (SST). IoMT-based SSD are those systems which assist with early diagnosis and tracing of the symptoms at the individual level while coupled with certain algorithms and additional hardware, SST technology are those technologies to model not only the individual symptoms but also the dynamics of symptom evolution in clusters of population based on interaction models and tracing of interpersonal interaction for better management of the spread in a cluster and on a larger scale in society.

In this perspective article, we disseminate our perspective about the challenges and potentials for the use of SSD technologies to continuously and autonomously monitor the vital signs of patients can be to alert the individual and the care providers about any upcoming potentially-major health anomalies so that proper medical care can be planned. We will discuss the imperative role of machine intelligence in particular health-related anomaly detection algorithms which can be used to not only detect but also predict the flares of symptoms. We will also highlight that how with the use of SSD technologies objective telemedicine sessions have been conducted, and how this can be further promoted to enhance telemedicine quality and reduce the need for in-person visits, and to avoid interpersonal contacts.

It should be noted that, continual monitoring allows for detecting infrequent flares of symptoms which may not be feasible based on infrequent discrete visits (Joyia et al., 2017; Khan et al., 2020). This is a major benefit of IoMT technologies which can significantly help with the fight against a pandemic, if low-cost, and highly-accessible wearable IoMT can be made available. This will not only help with a faster and more efficient assessment of the symptoms, but it also will help to distribute the healthcare resources optimally based on data collected from the affected patients. To further motivate more investment and investigation in this field it should be noted that SSD systems can also significantly help to monitor individuals before the infection and promote early diagnosis, planning, and management under remote access. This will be possible due to the available infrastructures for a smart and connected healthcare model which should be further enhanced to prepare the system for future waves of the pandemic and future pandemics.

The authors would like to emphasize their opinion that the use of IoMT devices can be extended to a higher level, for example, for clusters of patients in clinics or in small and then larger societies. This will be challenging but will allow monitoring not only the symptoms of individuals but also the spread of the symptoms. This concept has already been evaluated using smartphones in some couturiers (such as South Korea, India, Iceland) and some states in the United States (such as Utah), using GPS data of smartphones to monitor COVID-19 spread. However, GPS is not precise enough to gauge short distances, especially for in-door interactions. Thus, other forms of technologies such as Bluetooth Low-Energy (BLE) have been suggested (for example, by Google and Apple) on smartphones.

Based on the literature review conducted in this paper (explained later), despite the benefit of existing systems, such as BLE, the current technology has major limitations, among which we can highlight sensitivity to dynamic motions of the two carriers, sensitivity to a dynamic environment, the difficulty of calibration and need for re-calibration in a cluttered environment, and sensitivity to angle of arrival and location of the sender and receiver. This highlights a list of challenges that should be investigated for the higher performance of wearable IoMT on a large scale. Addressing these challenges, IoMT-based SST can implement preventive measures such as social distancing guidelines (SDG) based on the gathered multimodal information about (a) the symptoms evolution in a cluster of population and (b) interpersonal interaction in the clusters, especially in crowded indoor environments. Examples are medical facilities (such as dialysis clinics and neurorehabilitation clinics) and non-medical facilities, such as nursing homes, senior homes, drug rehabilitation facilities. On a larger scale, SST technology can enable medical providers to have a broader symptom monitoring over the society (and clusters of the population) in terms of the pandemic spread, and thereby manage the distribution of hospitalizations and medical supplies. It can be mentioned that the extended surveillance that SST technology grants can help policymakers to detect and react to the main causes of the spread by enacting more accurate laws to fight against the spread. SST technologies also raise awareness among the people about the dangerous areas of the city in regard to COVID-19 spread.

In this perspective article, we will also disseminate our opinion that both SST and SSD technologies can be embodied as a personal smart wearable device to help process the related bio-signals for diagnosis, tracking, and prevention. However, this would require significant optimization of electronics and investigation of means of reducing the cost to maximize accessibility and wide-use of such technology among the society regardless of the economic strength. This is a challenge to be addressed since most of the existing wearable systems either rely on connection with smartphones or have a very high cost, challenging the usability and feasibility of widely-used in societies with a low economy. In addition, despite all exciting benefits, data security, and reliability of data transmission can raise concerns and should be investigated thoroughly (Zhang Z. K. et al., 2014; Dorri et al., 2017; Khan and Salah, 2018; Noor and Hassan, 2019).

This article aims to initiate an in-depth conversation between different sectors, including researchers, technology designers, providers, hospitals, and policymakers to not only examine ways that can be implemented rapidly to adopt the existing technology and improve the health care system's diagnostic and preventative power using IoMTs but also to examine the challenges, and future directions of such technology in particular when the use is scaled-up to a societal level in order to fight possible future waves of COVID-19 pandemic and future pandemics. The authors would like to acknowledge that this article is written as a “perspective article type” to provide the opinion of the authors on the specific topic of the paper, i.e., the potentials of IoMT for COVID-19 response. Our intention in writing this article is to initiate discussions between researchers, policymakers, and stakeholders to further investigate the use of IoMT solutions for empowering the healthcare systems under the severe restrictions imposed by COVID-19 and considering the unfortunate current and future waves of this pandemic and future pandemics.

## 2. Internet of (Medical) Things in the Era of COVID19

IoMT has exponentially become more popular during the past decade due to the benefits for creating smart environments that can autonomously function to provide various services (Bélissent, 2010; Sundmaeker et al., 2010; Gubbi et al., 2013). IoMT wearable devices have been increasingly used for medical purposes, such as monitoring health of elderly (Liang and Yuan, 2016), physical activity monitoring (Wang and Tang, 2020), and orthopedic care (Singh et al., 2020). However, most of the pre-COVID-19 uses of IoMT devices were for small-scale application and in many cases when the cost and scalability were not an issue. Given the large-scale challenges caused by COVID-19 pandemic, autonomous services, and remote conduction of service (telepresence) have become of higher importance, in particular in the context of telemedicine (Singh et al., 2020) calling for large-scale use of affordable and accessible technology which can be used in remote areas and in regions with limited economic power. Several governmental funding agencies are now supporting research proposals across the world for designing low-cost scalable IoMT devices to enhance the health care system during the fight with COVID-19. Examples are funded NSF RAPID grants (Atashzar and Wang, 2020; Rogers, 2020), in addition to numerous calls for proposals, such as NRC (2020). This shows the imperative unmet need for having very low cost and effective IoMT devices for telemedicine which requires addressing a wide range of technical challenges including the accuracy, wearability, ease-of-use (specially for aged population) in unstructured dynamic environments and with minimum to no re-calibration needs. For this there is a need for discussing the building block of an IoMT framework in the context of COVID-19. IoMT frameworks are composed of two cores, namely, hardware and middleware (Gubbi et al., 2013).

### 2.1. Hardware

Hardware includes all the sensors that monitor biomarkers and symptoms. To choose the best sensors for tracking symptoms of COVID-19, first, we should have an in-depth insight into the symptoms of COVID-19 infection. Then we should choose the most appropriate sensors for tracking the symptoms, considering the cost for large scale deployment, need for calibration, re-calibration, and the ease of use in the context of a wearable system for the society.

Current identified symptoms of COVID-19 are predominantly fever (Huang et al., 2020; Roser et al., 2020; Wang D. et al., 2020), dry cough (Chen et al., 2020; Huang et al., 2020; Roser et al., 2020; Wang D. et al., 2020), fatigue (Huang et al., 2020; Roser et al., 2020; Wang D. et al., 2020), a drop of SpO2 with minimum signs (happy hypoxemia) (Guo et al., 2020; Tobin et al., 2020), and other symptoms that are less frequent, though can be more serious, e.g., shortness of breath (Chen et al., 2020; Roser et al., 2020), headache (Chen et al., 2020; Huang et al., 2020; Roser et al., 2020; Wang D. et al., 2020), and muscle pain (Chen et al., 2020; Roser et al., 2020).

Since COVID-19 is still known to be a respiratory disease, achieving information about blood oxygen saturation level is essential. It should be highlighted that happy hypoxemia is an unconventional situation because of which patients who have critical oxygen saturation do not feel unwell for a long period of time during which the infection gets worse, while patients do not show serious symptoms resulting in delayed delivery of care. This shows the importance of detecting such a condition as early as possible. Pulse oximeter sensors measure pulse rate and the level of oxygen saturation in reduced hemoglobin (Hb), based on the light absorption characteristic (Vandecasteele et al., 2017).

Challenges for using Spo2 is the sensitivity of such sensor to the contact quality and possibility blockage issues (such as due to body hair) affecting the reflective light, also sensitivity to movements. Thus choosing the best number of sensors (to conduct redundant recording) and the best location on the body are imperative topics to be investigated when designing the wearable for a large scale. One solution is to use the multichannel recording to reduce the chance of blockage and increase the signal to noise ratio by fusing the recording. However, this would increase the cost, size, and computational load. A comprehensive analysis is needed to test various locations on the body, which can provide a robust recording. The most successful wearable systems in the market are smartwatches. However, due to the complexity of the physiology at the wrist, recording SPo2 has been a major challenge for smartwatch companies. A limited number of very recent smartwatches in the marker offer SPo2 recording; however, they require a very steady posture for a prolonged duration, which will be a challenge for patients or elderly users. Also, these systems are not able to provide continual recording, limiting the chance of picking up the dynamic changes.

Another challenge of existing IoMT devices is the need for being paired with a smartphone. This significantly increases the cost and reduces accessibility, especially for remote areas and for areas with a low economy. Thus there is an unmet need for having an IoMT device that can not only accurately measure the symptoms but also be independent of any edge device and can operate as a stand-alone technology with minimum cost. As mentioned before, some grant agencies are calling for new proposals to generate stand-alone IoMT devices under 50$. Of course, the accuracy cannot be sacrificed, especially since the recordings are very sensitive. For example, a SPo2 of 91 out of 100 may require immediate attention, and this cannot be within the range of error of the hardware.

In addition to Spo2, respiratory rate (RR) can be achieved by various means, such as advanced processing of ECG (Shen et al., 2017) or through the use of an array of piezoresistive films placed non-invasively around an individual's chest to sense the frequency of the chest motion (Loriga et al., 2006; Pacelli et al., 2006a,b; Witt et al., 2011; Fiedler et al., 2012; Atalay et al., 2014; Subbe and Kinsella, 2018). The challenge with measuring RR is the very low-frequency content, which makes it computationally difficult to estimate based on bioelectrical recording such as ECG. There are also specific challenges with any bioelectric recording, as explained below. Using pressure belts can provide a measure of RR, but it would challenge the wearability and usability of the system and makes it difficult for large-scale uses. The topic of calculating RR is an accelerated field of research, and more recent efforts are focused on using other modalities (such as optical PPG) to extract RR.

Body temperature is the most important information for COVID-19 (Roser et al., 2020). In order to measure this modality, contact sensors and IR sensors have been used. IR-based temperature sensors provide better performance in rejecting the ambient noise and less sensitivity to contact conditions (Stavem et al., 1997; Liang and Yuan, 2016) when compared with contact sensors (Sibinski et al., 2010), thus it is suggested for smart wearables.

For detecting functionality of cardiovascular system besides symptoms of fatigue, muscle soreness, stress, and heart rate (and possibly RR), bioelectrical signals (such as EEG, EMG, and ECG) can be used as information rich markers (Gazendam and Hof, 2007; Jap et al., 2009; Craven et al., 2014; Rechy-Ramirez and Hu, 2015; Acharya et al., 2018; Xia et al., 2018). Bioelectrical recording however may face challenges such as being affected by the electromagnetic noise of the household devices, or changes in electrical impedance and connectivity stemming from sweating and other physiological causes. Substitutional sensing modalities have been used in wearable IoMT devices. For monitoring heart rate, PPG may replace ECG while relaxing the dependency to electrical contact, and for monitoring muscle activities, mechanomyography, or force-myography may replace EMG (Castillo et al., 2020). Besides sensors, communication, and power electronics are other modules of hardware in a wearable IoMT, the complexity of which depends on the bandwidth needed and power consumption.

From a communication standpoint, in wearable IoMT devices, near field connection (NFC) (Neefs et al., 2010; Opperman and Hancke, 2011; Timalsina et al., 2012; Duregger et al., 2015), Bluetooth connection (Lee et al., 2007; Dementyev et al., 2013), and WiFi (Lee et al., 2007; Curone et al., 2010; Kim et al., 2015) are used based on their data transfer rate, range of communication capability, power consumption, and availability. Some of these communication modalities are also used for localization, as explained later. It should be added that the communication module of wearable systems has been seen as a potential solution for addressing the contact tracing problem. For example, there is a wide range of studies on the use of Bluetooth low energy. Later in this document, we provide our perspectives on the benefits and challenges of the use of such a solution for detecting interpersonal contact between the wearers.

Thus, it can be mentioned that despite a wide range of available sensing technologies, particular investigations are needed to minimize the cost while maximizing the accuracy and wearability. In the above, a range of challenges with existing technologies is provided, which shows the roadmap that can be taken to realize a scalable solution.

### 2.2. Middleware

Middleware administrates storing the information and evaluating the collected data to extract meaningful features that can be assessed on the fly to (a) provide biofeedback to the user (Sundmaeker et al., 2010), and (b) provide information from a cluster of users for analysis by medical workers, policymakers, and other public sectors, which helps to monitor the effects of healthcare and guidelines at the societal level. There are different architectures of IoMT middleware that are utilized based on the expected functionality of the framework. These architectures can be categorized mainly as service-based (Papazoglou and Georgakopoulos, 2003), cloud-based (Ngu et al., 2016), and actor-based (Soldatos et al., 2015) modules. As a part of middleware, diagnostic IoMT technologies can be equipped with means of artificial intelligence to predict health-related anomalies. In the rest of this article, we provide our perspective on this important topic as well.

#### 2.2.1. Security and Privacy

Privacy is a significant concern and must be addressed before any potential large-scale use of IoMT devices for contact tracing and symptom tracking. Without a systematic solution which provides a very high degree of protection on patient's data, IoMT devices can only be used up to a limited scale, such as for in a hospital uses or for clusters of the high-risk population in a closed space (such as nursing homes to track symptom evolution in the population), or as part of telemedicine and individual uses. These are examples of limited scale uses for which the important matter of privacy and security can be addressed using existing infrastructures. For any large-scale use of the device, a serious concern that needs to be addressed is the matter of large scale security and privacy of the information. Relevant important discussions can be found in Subashini and Kavitha (2011), Zhang Z. K. et al. (2014), Dorri et al. (2017), Gatouillat et al. (2018), Khan and Salah (2018), Hatzivasilis et al. (2019), Noor and Hassan (2019), and Kagita et al. (2020).

The authors also believe that one additional issue related to this topic is the reliability of data storage and data transmission and accessibility of the medical sector to such data. Since internet-based architectures that handle personal information can be a subject of different attacks, there is an imperative need for utilizing security algorithms. Examples can be found in the literature focusing on the maintenance of the safety of such systems (Sicari et al., 2015). In addition to compromising information confidentiality, large-scale uses of IoMT architectures can increase the susceptibility to malicious cyber-physical attacks that are aiming to hinder the processing of the data and causing failures, false-positive alarms, and false-negative reports (Khan and Salah, 2018). These attacks can range from low-level (Xu et al., 2005) to intermediate-level (Zhang K. et al., 2014) and high-level (Conzon et al., 2012). For addressing this issue, there is a need for implementing defense mechanisms. Several defense techniques have been proposed in the literature for each type of attack, which should be investigated before a large-scale IoMT can be deployed (Xiao et al., 2009; Bhattasali and Chaki, 2011; Khan and Salah, 2018).

## 3. IoMT Wearable Technologies

Due to the potential benefit of IoMT devices, there have been an accelerated range of recent efforts that envision the use for fighting against COVID-19 spread and future pandemics. These aim at the conduction of early diagnosis, tracking the spread, and monitoring the infected and susceptible individuals (Atashzar and Wang, 2020; Dong et al., 2020; Garg, 2020; Roser et al., 2020; Sohrabi et al., 2020; Wu and McGoogan, 2020). The trend (Ng et al., 2020) is motivated with the imperative need to prevent the spread of the COVID-19 on different societal levels. In order to discuss various functionality of IoMT wearable technologies, in this prespective article, authors have categorized IoMT wearables into SSD and SST.

### 3.1. Systems for Symptom Decoding

Technologies, which are called Systems for Symptom Decoding (SSD) in this paper, are designed for diagnosis, monitoring, analyzing the evolution of signs of infection at an individual level. Upon achieving the biomarkers via the hardware in an SSD, the information can be sent to the (cloud-based) middleware to be processed using various AI-based anomaly detection algorithms, which are machine learning modules that process the distribution of multidimensional data and detect health-related anomalies.

The authors would like to highlight that based on conventional machine-learning-based anomaly detection approaches, subtle multidimensional changes in the well-being of an individual can be tracked to inform the medical correspondent about the malevolent alterations in the biomarkers, to promote early diagnosis of COVID-19 infection, fighting the prolonged incubation period of COVID-19 (Zhai et al., 2020).

In terms of the type of algorithm for detecting infection-related health anomalies, gray box and black box artificial intelligence models can be used (see examples in the literature; Khan and Khan, 2012), some of which rely on probabilistic distributions of the data, and some rely on underlying labeled patterns in the healthy data to be modeled. Based on probabilistic algorithms, the likelihood of infection for an individual can be calculated. In this regard, we can highlight two main subcategories for health-related anomaly detection, which can be used in IoMT wearable for COVID-19, namely (a) clustering-based techniques, (b) classification-based techniques.

In this regard, the K-means clustering approach (Tan et al., 2016), K-medoids approach (Garg et al., 2020), and Expectation-Maximization-based clustering approaches such as mixture models (Bublitz et al., 2017; Qi et al., 2018) are among the candidates for clustering techniques. These machine learning modules try to detect the underlying clusters of multidimensional data and predict an anomaly if the new data does not show a high probability of belonging to one of the clusters.

In addition, the Fuzzy logic approach (Hamamoto et al., 2018), genetic algorithm (Chen et al., 2018), naïve Bayes networks (Zhen et al., 2017), neural networks (Amarasinghe et al., 2018; Chalapathy and Chawla, 2019), and support vector machines (Erfani et al., 2016) are among classification algorithms used for anomaly detection, which can be used for detecting COVID-19 anomalies in the symptom space of patients.

The authors' perspective about the context of detecting the health anomalies of COVID-19 based on multidimensional data collected by wearables is as follows. Despite the great success and advancements in the field, the anomaly detection algorithms suffer from several issues which are pronounced for COVID-19, including (a) the sensitivity of the accuracy to the amount of available labeled data (this is concerning in the context of COVID-19 since the data is limited due to the novelty of the virus and limited knowledge and consistent data collection), (b) variation in the normal behavior of the data and the definition of normal behavior (which is questionable due to the very different and unpredictable behavior of the virus for different individuals), (c) noise in data (which is a challenge for any wearable and in-home technologies), (d) similarity between advanced anomalies and normal data (which is a problem due to heterogeneity of symptoms of COVID-19). Some of the aforementioned concerns are rooted in existing issues of health-related and more information in this regard can be found in Papazoglou and Georgakopoulos (2003), Xu et al. (2005), Xiao et al. (2009), Bhattasali and Chaki (2011), Conzon et al. (2012), Zhang K. et al. (2014), Agrawal and Agrawal (2015), Rechy-Ramirez and Hu (2015), Sicari et al. (2015), Soldatos et al. (2015), Ngu et al. (2016), and Khan and Salah (2018).

Our perspective about the near future of AI-based diagnostic techniques for COVID-19 and other infectious disease is that the SSD technologies can be augmented by algorithms which can facilitate prediction-in-time (beyond monitoring) the “evolution” of symptom biosignals. This can significantly augment the versatility of the system. In this regard, machine learning algorithms that can predict a possible near future adverse event over a given prediction horizon can be significantly beneficial as it would allow for early diagnosis and planning. The longer the prediction horizon, the more complex yet more beneficial the algorithms will be. This is a challenging task; however, the authors believe that it can be realized in the near future using state of the art neural network architectures, specifically LSTM or GRU (which are two modern formats of recurrent neural networks for processing time series). However, these models are supervised techniques and require heavy data collection. A new variant of neural network architecture that can help with addressing this issue is shallow neural networks. Thus, the authors believe that a combination of a shallow neural network and a recurrent neural network architecture can provide the needed temporal resolution in terms of the prediction horizon for diagnosing infection for COVID-19 symptoms. The use of shallow architectures reduces the need for heavy data collection, and the use of advanced modules such as GRU, which is designed to be more efficient, allows for underlying modeling patterns of symptom evolution that can be decoded for early prediction of infection progress.

**Figure 1:**
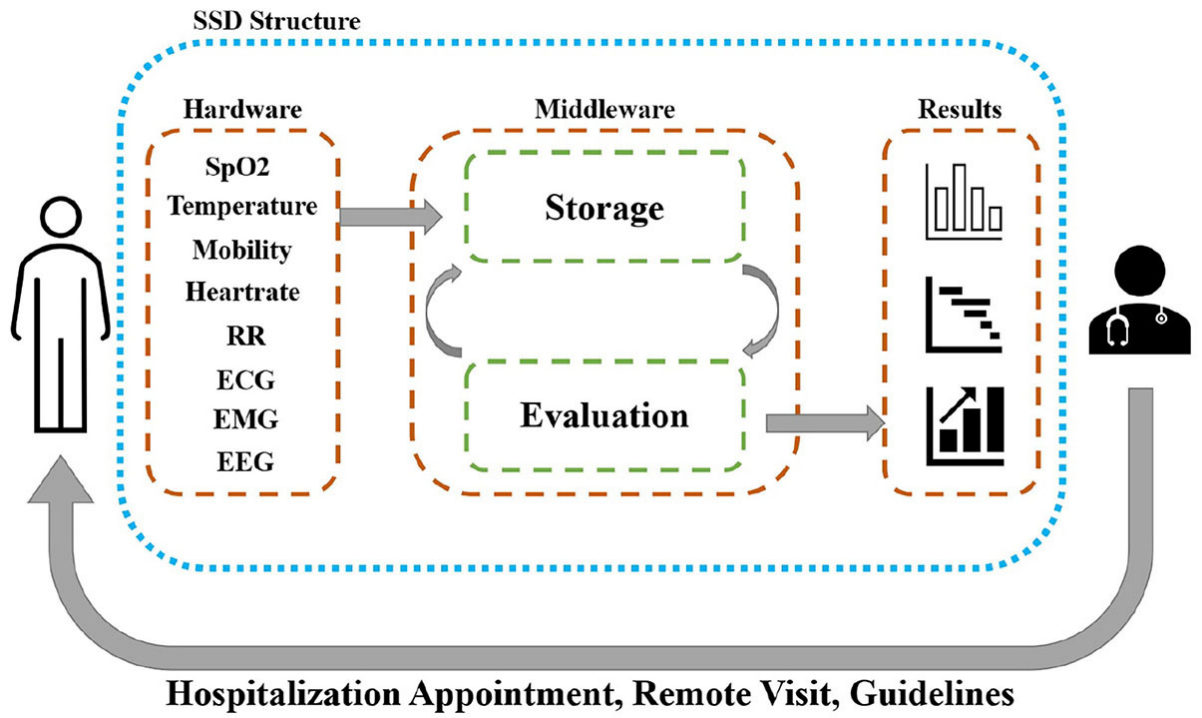
Functionality of SSD.

Thus, it can be summarized that thanks to the advances in the last decade on neural network architectures, the next generation of wearable IoMT devices can be augmented with cloud computation allowing for accessing strong machine intelligence for early detection and possibly prediction (with a tunable horizon) for health-related anomalies. However, this requires widespread and fast access to cloud computation infrastructure. There exists a rich literature for detecting general health anomalies to be adopted in COVID-19 IoMT wearables; however, there exist several challenges that should be investigated, as discussed in the above. This would call for investment and investigation to empower wearable technologies of tomorrow with means of predictive diagnosis intelligence. This can significantly enhance the protocols and diagnostic workflow. For example, results of the anomaly assessment can be forwarded to the medical correspondent to accordingly schedule hospitalization and online visits or suggest guidelines to the possibly infected person. Figure 1 shows the overall concept of SSD.

### 3.2. Systems for Spread Tracing

In this paper, we categorize SST as technologies that will take the analyzed information from multiple SSD systems to monitor the aggregation of information from a cluster of users to assess the current status of the spread of COVID-19 and suggest guidelines for the communities (including users and non-users of SSDs) to help avoid the contraction of the COVID-19 especially for high-risk populations and plan for minimizing the risk of infections for non-affected groups.

The authors would like to highlight that SST can be identified as the more general IoMT surveillance system for a population cluster as it evaluates both medical biomarkers (those collected by SSD) and non-medical information regarding the interpersonal interaction between individuals. As an example, our perspective is that in an in-patient non-COVID clinic in a hospital where there are clusters of patients, clinicians, and visitors, deployment of an SST technology (such as smart tags) can allow for monitoring the evolution of symptoms among the under surveillance population to minimize the risk of confrontation and detecting early spread and hotspots of infection. This will be imperative for (a) controlling the spread, (b) isolating the non-infected individuals, and (c) planning for implementation of a more efficient SDG.

It is of high importance to track and backtrace the path that led to an infection, to monitor the early or recovered cases, and to collect data for future analyses. This requires significant human resources, clinical resources, and time which are all in shortage currently in healthcare systems (Boulos and Geraghty, 2020; Dong et al., 2020; Emanuel et al., 2020; Fauci et al., 2020; Menni et al., 2020). Here, “tracking” is defined as gathering information about (a) the history of an individual's locations, (b) people that the individual has visited, and (c) tracking back to the infection source. The authors would like to highlight that currently, still in many couturiers (not all), this process has been done by subjective surveys, which are very costly, non-objective, time-consuming, and not necessarily accurate as in many cases, an individual in the chain of interaction may have mild or happy symptoms (which exist but are not felt as mentioned before). This shows the importance of objective tracking of the trace of the virus by (a) collecting multidimensional symptom markers and (b) history of interaction, and (c) compliance to the SDG. This topic is discussed in detail in section 3.2, and the authors have introduced recent efforts by industries such as Google and Apple and some governments to use advanced technologies such as GPS and BLE to promote objective contact tracking using smartphone technologies. This highlights the ongoing accelerated effort, which further supports the use of wearable IoMT technologies (equipped with contact tracking technologies) for COVID-19.

It should be noted that the benefit of augmenting sensorized wearable technologies with biomarker and contact tracking features is that the technology is able to not only track the location but also concurrently the symptoms of the user to generate a better model of infection spread in society and to better protect the wearer and the visitors to the hotspots and estimate the infection severity in various regions.

However, we should highlight that there is a wide range of technical bottlenecks. Among the existing challenges, we would light to highlight (a) localization accuracy and resolution in a dynamic, unstructured, and cluttered environment and (b) the security and privacy of the wearers. Regarding outdoor localization, it can be mentioned that although GPS accuracy may not be at the ideal level to detect interpersonal interaction, it is sufficient for detecting whether an individual has been in a crowded or infected hotspot zone or if a region is showing flares of symptoms. It can also be used to detect whether an individual has been following SDG. The use of such an advanced approach allows for the generation of density heatmaps of the cluster of crowds and that of symptoms and analysis of the interaction between the two clusters.

Regarding indoor localization, however, the state of the art techniques are designed based on the use of Bluetooth Low Energy (BLE) (Ng et al., 2020; Sadowski et al., 2020; Spachos and Plataniotis, 2020a,b), Ultra High-Frequency RFID (Li et al., 2019), WiFi (Wang et al., 2017), and hybrid systems (Guo et al., 2019; Monica and Bergenti, 2019). Our perspective is that using these technologies; a wearable IoMT device can be equipped with cloud-based signal triangulation techniques and advanced filtering, data fusion, and estimation approaches (such as Kalman-based sensor fusion and machine learning techniques) to locate the wearer with respect to the known locations of signal transmitters installed in an indoor infrastructure. The technical challenges are (a) accuracy needed for detecting interpersonal interactions, (b) high sensitivity to dynamical movements within the unstructured under-surveillance environment and movement artifacts from the wearers, (c) the cost of the systematic infrastructure needed for signal triangulation, and (d) patient privacy.

**Figure 2:**
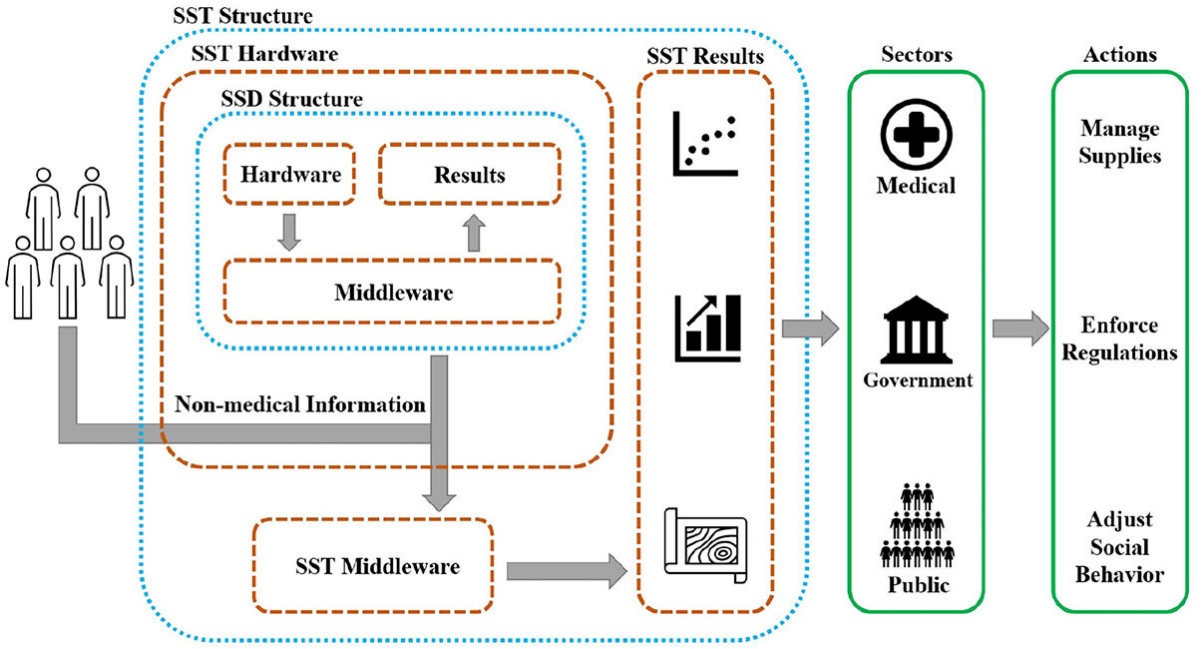
Functionality of SST.

The authors would like to highlight that with the use of SST, it can be inferred if an under-surveillance society is following the preventive guidelines and how the symptom activity is spreading among the population. In addition, we believe that optimizing the effort to treat hotspots detected by wearable systems can help the policymakers to reevaluate the regulations based on the real-time status of symptom spread. Thus it can be mentioned that SST can help the sectors in charge to smartly alter the intensity of the public regulations for controlling the COVID-19 spread to manage the spatiotemporal aspect of the reopening process while ascertaining the public compliance with preventive guidelines. The authors' opinion is that the use of wearable technologies can help to better predict upcoming waves in various zones and to objectively plan for sourcing medical supplies to avoid urgent shortages. As an additional feature, symptom activities in various clusters can be shared on a common platform with the society to let commuters avoid facing hotspots with a higher risk of infection. Figure 2 shows a schematic view of SST's functionality.

The authors would like to highlight that the topic of contact tracing using advanced proximity sensing, such as using Bluetooth low energy (BLE) technology, is an active field of research and recently is more accelerated due to the benefit of tracking and backtracing contacts between individuals with a positive history of COVID-19 infection and other users of the technology. The use of BLE is motivated due to the availability of it in smartphones and because of the functionality for indoor locations to track interpersonal contacts. In this regard, it should be noted that some governments are suggesting the use of this technology for current waves of the pandemic. As mentioned in Servick (2020) (the following quoted text is taken from Servick, 2020); currently, “GPS data from phones can identify potential hot spots and indicate who has been exposed. Government programs in South Korea, India, Iceland, and U.S. states, including North Dakota and Utah, are using phone location data to monitor COVID-19 spread. But GPS technology is not precise enough to gauge short distances between two phones to determine which encounters are most risky.” This is the motivation for using other platforms such as BLE to detect interpersonal interaction. Please see more details in Ng et al. (2020), Servick (2020), and Zastrow (2020) for an explanation of the use of BLE technology on smartphone applications for tracking COVID-19 infection and some recent efforts such as those by Google and Apple for releasing an application for doing this. Also please see Wang et al. (2017), Guo et al. (2019), Li et al. (2019), Monica and Bergenti (2019), Mackey et al. (2020), Sadowski et al. (2020), Spachos and Plataniotis (2020a), and Spachos and Plataniotis (2020b) for more details on technologies that can be used for indoor tracking. However, there is a wide range of challenges to be addressed for the use of such technology. The challenges are mainly related to the achievable accuracy of such a technique for an unstructured, cluttered, and dynamic environment. Also, the need for calibration, the sensitivity to angle of arrival, and the location of the sensor, and the motion of the wearer further challenge the use of such a system. The current efforts are toward (a) developing new machine intelligence algorithms to further enhance the accuracy of the system, (b) fusion with other modalities while keeping the cost low to enhance the resolution.

The authors believe that the wearable technologies of tomorrow will be able to estimate social distancing without reliance on communication with smartphones to minimize the cost and maximize accessibility while providing the needed accuracy and resolution. This calls for an extensive investigation and investment related to the field of IoMT wearables and can significantly reform the future of the modern healthcare system through more objective telemedicine.

It should be noted that a situation that can potentially challenge this technology is the large-scale acceptability of the society for the use of the proposed approach. The high-scale use can be affected by the resistance of different groups for the adoption of this technology and the lack of compliance. These are open challenges facing large-scale use of any new technology, which may initially limit the feasibility at the societal level. A gradual adoption may be suggested starting from smaller populations such as people in nursing homes and those with co-morbidities, then scaling it to higher volumes. The aforementioned challenges call for an active discussion with and involvement of social scientists and policymakers, who can help to investigate the underlying reasons for the potential rejection of large-scale uses, and thus implement the needed training and deliver accurate information to allow for a higher volume of use and higher compliance at the societal level.

## 4. Conclusion

In this paper, the authors disseminate their perspective on the use, functionality, and challenges of Wearable IoMT technologies coupled with artificial intelligence for changing the picture of telehealth during a global pandemic in which remoteness, cost, accessibility, efficacy, and versatility are crucial to managing the infection symptoms at the individual level, in clusters of high-risk populations, and ultimately in society. The authors believe that to deploy this technology and benefit from its multifaceted objective features, and several sectors should be informed and agree on terms of operation. This perspective article aims at providing insight on various aspects of wearable IoMT, elucidating existing advances and challenges while highlighting the potential benefit for managing the future waves of COVID-19 pandemic and future pandemics. We emphasize how this technology can help to conduct early diagnosis at individual levels and how it can help with optimizing the governmental regulations based on the interaction between high-risk population clusters and symptom spread. This article aims at increasing the awareness of the society, governments, medical correspondents, and industries about this new smart way of surveillance of infection and spread to act accordingly by enacting regulatory laws, providing medical supports, optimizing plans for testing and hospitalization, and monitoring the compliance. There are several technical and technological challenges to be addressed, listed in this paper, calling for extensive investigation and investment on the topic of IoMT Wearable Technologies.

## Data Availability Statement

The original contributions presented in the study are included in the article/supplementary material, further inquiries can be directed to the corresponding author/s.

## Author Contributions

SM, YW, and SA collaborated on the conceptualization of this perspective article, conducting the literature review and demographic study, analyzing the existing technologies, and writing and editing the paper. All authors contributed to the article and approved the submitted version.

## Conflict of Interest

The authors declare that the research was conducted in the absence of any commercial or financial relationships that could be construed as a potential conflict of interest.

## References

[B1] Acharya U. R. Oh S. L. Hagiwara Y. Tan J. H. Adeli H. (2018). Deep convolutional neural network for the automated detection and diagnosis of seizure using EEG signals. Comput. Biol. Med. 100, 270–278. 10.1016/j.compbiomed.2017.09.017 28974302

[B2] Adhikari S. P. Meng S. Wu Y.-J. Mao Y.-P. Ye R.-X. Wang Q.-Z.. (2020). Epidemiology, causes, clinical manifestation and diagnosis, prevention and control of coronavirus disease (COVID-19) during the early outbreak period: a scoping review. Infect. Dis. Pov. 9, 1–12. 10.1186/s40249-020-00646-x 32183901 PMC7079521

[B3] Agrawal S. Agrawal J. (2015). Survey on anomaly detection using data mining techniques. Proc. Comput. Sci. 60, 708–713. 10.1016/j.procs.2015.08.220

[B4] Ainslie K. E. Walters C. E. Fu H. Bhatia S. Wang H. Xi X.. (2020). Evidence of initial success for china exiting COVID-19 social distancing policy after achieving containment. Wellcome Open Res. 5, 1–14. 10.12688/wellcomeopenres.15843.2 32500100 PMC7236587

[B5] Amarasinghe K. Kenney K. Manic M. (2018). Toward explainable deep neural network based anomaly detection, in 2018 11th International Conference on Human System Interaction (HSI) (Gdansk), 311–317. 10.1109/HSI.2018.8430788

[B6] Atalay O. Kennon W. R. Demirok E. (2014). Weft-knitted strain sensor for monitoring respiratory rate and its electro-mechanical modeling. IEEE Sens. J. 15, 110–122. 10.1109/JSEN.2014.2339739

[B7] Atashzar S. F. Wang Y. (2020). NSF Rapid: SCH: Smart Wearable COVID19 Biotracker Necklace: Remote Assessment and Monitoring of Symptoms for Early Diagnosis, Continual Monitoring, and Prediction of Adverse Event. Alexandria, VI: US National Science Foundation. Available online at: https://www.nsf.gov/awardsearch/showAward?AWD_ID=2031594&HistoricalAwards=false

[B8] Bélissent J. (2010). Getting Clever About Smart Cities: New Opportunities Require New Business Models. (Cambridge, MA), 244–277.

[B9] Bhattasali T. Chaki R. (2011). A survey of recent intrusion detection systems for wireless sensor network, in International Conference on Network Security and Applications (Chennai: Springer), 268–280. 10.1007/978-3-642-22540-6_27

[B10] Boulos M. N. K. Geraghty E. M. (2020). Geographical tracking and mapping of coronavirus disease COVID-19/severe acute respiratory syndrome coronavirus 2 (SARS-CoV-2) epidemic and associated events around the world: how 21st century GIS technologies are supporting the global fight against outbreaks and epidemics. Int. J. Health Geogr. 19:8. 10.1186/s12942-020-00202-8 32160889 PMC7065369

[B11] Bublitz C. F. Ribeiro-Teixeira A. C. Pianoschi T. A. Rochol J. Both C. B. (2017). Unsupervised segmentation and classification of snoring events for mobile health, in GLOBECOM 2017-2017 IEEE Global Communications Conference (Singapore), 1–6. 10.1109/GLOCOM.2017.8255031

[B12] Castillo C. S. M. Atashzar S. F. Vaidyanathan R. (2020). 3D-mechanomyography: accessing deeper muscle information non-invasively for human-machine interfacing, in 2020 IEEE/ASME International Conference on Advanced Intelligent Mechatronics (AIM) (Boston, MA), 1458–1463. 10.1109/AIM43001.2020.9159036

[B13] Chalapathy R. Chawla S. (2019). Deep learning for anomaly detection: a survey. arXiv preprint arXiv:1901.03407.

[B14] Chen N. Zhou M. Dong X. Qu J. Gong F. Han Y.. (2020). Epidemiological and clinical characteristics of 99 cases of 2019 novel coronavirus pneumonia in Wuhan, china: a descriptive study. Lancet 395, 507–513. 10.1016/S0140-6736(20)30211-7 32007143 PMC7135076

[B15] Chen S. Wen P. Zhao S. Huang D. Wu M. Zhang Y. (2018). A data fusion-based methodology of constructing health indicators for anomaly detection and prognostics, in 2018 International Conference on Sensing, Diagnostics, Prognostics, and Control (SDPC) (Xi'an), 570–576. 10.1109/SDPC.2018.8664723

[B16] Conzon D. Bolognesi T. Brizzi P. Lotito A. Tomasi R. Spirito M. A. (2012). The virtus middleware: an XMPP based architecture for secure IOT communications, in 2012 21st International Conference on Computer Communications and Networks (ICCCN) (Munich), 1–6. 10.1109/ICCCN.2012.6289309

[B17] Craven D. McGinley B. Kilmartin L. Glavin M. Jones E. (2014). Compressed sensing for bioelectric signals: a review. IEEE J. Biomed. Health Inform. 19, 529–540. 10.1109/JBHI.2014.2327194 24879647

[B18] Curone D. Secco E. L. Tognetti A. Loriga G. Dudnik G. Risatti M.. (2010). Smart garments for emergency operators: the proetex project. IEEE Transactions on Information Technology in Biomed 14, 694-701. 10.1109/TITB.2010.2045003 20371413

[B19] Dementyev A. Hodges S. Taylor S. Smith J. (2013). Power consumption analysis of bluetooth low energy, zigbee and ant sensor nodes in a cyclic sleep scenario, in 2013 IEEE International Wireless Symposium (IWS) (Beijing), 1–4. 10.1109/IEEE-IWS.2013.6616827

[B20] Dong E. Du H. Gardner L. (2020). An interactive web-based dashboard to track COVID-19 in real time. Lancet Infect. Dis. 20, 533–534. 10.1016/S1473-3099(20)30120-1 32087114 PMC7159018

[B21] Dorri A. Kanhere S. S. Jurdak R. Gauravaram P. (2017). Blockchain for IOT security and privacy: The case study of a smart home, in 2017 IEEE International Conference on Pervasive Computing and Communications Workshops (PerCom Workshops) (Kona, HI), 618–623. 10.1109/PERCOMW.2017.7917634

[B22] Duregger K. Hayn D. Morak J. Ladenstein R. Schreier G. (2015). An mHealth system for toxicity monitoring of paediatric oncological patients using near field communication technology, in 2015 37th Annual International Conference of the IEEE Engineering in Medicine and Biology Society (EMBC) (Milan), 6848–6851. 10.1109/EMBC.2015.7319966 26737866

[B23] Emanuel E. J. Persad G. Upshur R. Thome B. Parker M. Glickman A.. (2020). Fair allocation of scarce medical resources in the time of Covid-19. N. Engl. J. Med. 382, 2049–2055. 10.1056/NEJMsb2005114 32202722

[B24] Erfani S. M. Rajasegarar S. Karunasekera S. Leckie C. (2016). High-dimensional and large-scale anomaly detection using a linear one-class SVM with deep learning. Pattern Recogn. 58, 121–134. 10.1016/j.patcog.2016.03.028

[B25] Fauci A. S. Lane H. C. Redfield R. R. (2020). Covid-19-navigating the uncharted. N. Engl. J. Med. 382, 1268–1269. 10.1056/NEJMe2002387 32109011 PMC7121221

[B26] Fiedler P. Biller S. Griebel S. Haueisen J. (2012). Impedance pneumography using textile electrodes, in 2012 Annual International Conference of the IEEE Engineering in Medicine and Biology Society (San Diego, CA), 1606–1609. 10.1109/EMBC.2012.6346252 23366213

[B27] Garg S. (2020). Hospitalization Rates and Characteristics of Patients Hospitalized With Laboratory-Confirmed Coronavirus Disease 2019-COVID-Net, 14 States, March 1-30, 2020. MMWR. Morbidity and Mortality Weekly Report, 69. Centers for Disease Control and Prevention. 32298251 10.15585/mmwr.mm6915e3 PMC7755063

[B28] Garg S. Kaur K. Batra S. Kaddoum G. Kumar N. Boukerche A. (2020). A multi-stage anomaly detection scheme for augmenting the security in iot-enabled applications. Fut. Gener. Comput. Syst. 104, 105–118. 10.1016/j.future.2019.09.038

[B29] Gatouillat A. Badr Y. Massot B. Sejdić E. (2018). Internet of medical things: a review of recent contributions dealing with cyber-physical systems in medicine. IEEE Intern. Things J. 5, 3810–3822. 10.1109/JIOT.2018.2849014

[B30] Gazendam M. G. Hof A. L. (2007). Averaged EMG profiles in jogging and running at different speeds. Gait Posture 25, 604–614. 10.1016/j.gaitpost.2006.06.013 16887351

[B31] Gubbi J. Buyya R. Marusic S. Palaniswami M. (2013). Internet of things (IOT): a vision, architectural elements, and future directions. Fut. Gener. Comput. Syst. 29, 1645–1660. 10.1016/j.future.2013.01.010

[B32] Guo G. Chen R. Ye F. Peng X. Liu Z. Pan Y. (2019). Indoor smartphone localization: a hybrid wifi RTT-RSS ranging approach. IEEE Access 7, 176767–176781. 10.1109/ACCESS.2019.2957753

[B33] Guo L. Ren L. Yang S. Xiao M. Chang D. Yang F.. (2020). Profiling early humoral response to diagnose novel coronavirus disease (COVID-19). Clin. Infect. Dis. 71, 778–785. 10.1093/cid/ciaa310 32198501 PMC7184472

[B34] Hamamoto A. H. Carvalho L. F. Sampaio L. D. H. Abrão T. Proença M. L. Jr. (2018). Network anomaly detection system using genetic algorithm and fuzzy logic. Expert Syst. Appl. 92, 390–402. 10.1016/j.eswa.2017.09.013

[B35] Hatzivasilis G. Soultatos O. Ioannidis S. Verikoukis C. Demetriou G. Tsatsoulis C. (2019). Review of security and privacy for the internet of medical things (IoMT), in 2019 15th International Conference on Distributed Computing in Sensor Systems (DCOSS) (Santorini), 457–464. 10.1109/DCOSS.2019.00091

[B36] Hellewell J. Abbott S. Gimma A. Bosse N. I. Jarvis C. I. Russell T. W.. (2020). Feasibility of controlling COVID-19 outbreaks by isolation of cases and contacts. Lancet Glob. Health 8, E488–E496. 10.1016/S2214-109X(20)30074-7 32119825 PMC7097845

[B37] Huang C. Wang Y. Li X. Ren L. Zhao J. Hu Y.. (2020). Clinical features of patients infected with 2019 novel coronavirus in Wuhan, china. Lancet 395, 497–506. 10.1016/S0140-6736(20)30183-5 31986264 PMC7159299

[B38] Jap B. T. Lal S. Fischer P. Bekiaris E. (2009). Using EEG spectral components to assess algorithms for detecting fatigue. Expert Syst. Appl. 36, 2352–2359. 10.1016/j.eswa.2007.12.043

[B39] Joyia G. J. Liaqat R. M. Farooq A. Rehman S. (2017). Internet of medical things (IoMT): applications, benefits and future challenges in healthcare domain. J. Commun. 12, 240–247. 10.12720/jcm.12.4.240-247

[B40] Kagita M. K. Thilakarathne N. Gadekallu T. R. Maddikunta P. K. R. (2020). A review on security and privacy of internet of medical things. arXiv preprint arXiv:2009.05394.

[B41] Khan M. A. Salah K. (2018). IoT security: review, blockchain solutions, and open challenges. Fut. Gener. Comput. Syst. 82, 395–411. 10.1016/j.future.2017.11.022

[B42] Khan M. E. Khan F. (2012). A comparative study of white box, black box and grey box testing techniques. Int. J. Adv. Comput. Sci. Appl. 3, 12–15. 10.14569/IJACSA.2012.030603

[B43] Khan S. R. Sikandar M. Almogren A. Din I. U. Guerrieri A. Fortino G. (2020). Iomt-based computational approach for detecting brain tumor. Fut. Gener. Comput. Syst. 109, 360–367. 10.1016/j.future.2020.03.054

[B44] Kim Y. Lee S. Lee S. (2015). Coexistence of Zigbee-based WBAN and Wifi for health telemonitoring systems. IEEE J. Biomed. Health Inform. 20, 222–230. 10.1109/JBHI.2014.2387867 25576586

[B45] Le T. T. Andreadakis Z. Kumar A. Roman R. G. Tollefsen S. Saville M.. (2020). The COVID-19 vaccine development landscape. Nat. Rev. Drug Discov. 19, 305–306. 10.1038/d41573-020-00151-8 32273591

[B46] Lee J.-S. Su Y.-W. Shen C.-C. (2007). A comparative study of wireless protocols: bluetooth, UWB, Zigbee, and Wi-fi, in IECON 2007-33rd Annual Conference of the IEEE Industrial Electronics Society (Taipei), 46–51. 10.1109/IECON.2007.4460126

[B47] Leung K. Wu J. T. Liu D. Leung G. M. (2020). First-wave COVID-19 transmissibility and severity in china outside HUBEI after control measures, and second-wave scenario planning: a modelling impact assessment. Lancet. 395, 1382–1393. 10.1016/S0140-6736(20)30746-7 32277878 PMC7195331

[B48] Li C. Mo L. Zhang D. (2019). Review on uhf RFID localization methods. IEEE J. Radio Freq. Identif. 3, 205–215. 10.1109/JRFID.2019.2924346

[B49] Li X. Geng M. Peng Y. Meng L. Lu S. (2020). Molecular immune pathogenesis and diagnosis of COVID-19. J. Pharm. Anal. 10, 102–108. 10.1016/j.jpha.2020.03.001 32282863 PMC7104082

[B50] Li Y. Xia L. (2020). Coronavirus disease 2019 (COVID-19): role of chest CT in diagnosis and management. Am. J. Roentgenol. 214, 1280–1286. 10.2214/AJR.20.22954 32130038

[B51] Li Y. Yao L. Li J. Chen L. Song Y. Cai Z.. (2020). Stability issues of RT-PCR testing of SARS-CoV-2 for hospitalized patients clinically diagnosed with COVID-19. J. Med. Virol. 92, 903–908. 10.1002/jmv.25786 32219885 PMC7228231

[B52] Liang T. Yuan Y. J. (2016). Wearable medical monitoring systems based on wireless networks: a review. IEEE Sens. J. 16, 8186–8199. 10.1109/JSEN.2016.259731223202028

[B53] Liu F. Xu A. Zhang Y. Xuan W. Yan T. Pan K.. (2020). Patients of COVID-19 may benefit from sustained lopinavir-combined regimen and the increase of eosinophil may predict the outcome of COVID-19 progression. Int. J. Infect. Dis. 95, 183–191. 10.1016/j.ijid.2020.03.013 32173576 PMC7193136

[B54] Long C. Xu H. Shen Q. Zhang X. Fan B. Wang C.. (2020). Diagnosis of the coronavirus disease (COVID-19): rRT-PCR or CT? Eur. J. Radiol. 2020:108961. 10.1016/j.ejrad.2020.108961 32229322 PMC7102545

[B55] Loriga G. Taccini N. De Rossi D. Paradiso R. (2006). Textile sensing interfaces for cardiopulmonary signs monitoring, in 2005 IEEE Engineering in Medicine and Biology 27th Annual Conference (Shanghai), 7349–7352. 10.1109/IEMBS.2005.1616209 17281978

[B56] Mackey A. Spachos P. Song L. Plataniotis K. N. (2020). Improving BLE beacon proximity estimation accuracy through Bayesian filtering. IEEE Intern. Things J. 7, 3160–3169. 10.1109/JIOT.2020.2965583

[B57] Menni C. Valdes A. M. Freidin M. B. Sudre C. H. Nguyen L. H. Drew D. A.. (2020). Real-time tracking of self-reported symptoms to predict potential COVID-19. Nat. Med. 26, 1037–1040. 10.1038/s41591-020-0916-2 32393804 PMC7751267

[B58] Monica S. Bergenti F. (2019). Hybrid indoor localization using Wifi and UWB technologies. Electronics 8:334. 10.3390/electronics8030334

[B59] Neefs J. Schrooyen F. Doggen J. Renckens K. (2010). Paper ticketing vs. electronic ticketing based on off-line system'tapango', in 2010 Second International Workshop on Near Field Communication (Monaco), 3–8. 10.1109/NFC.2010.24

[B60] Ng P. C. Spachos P. Plataniotis K. (2020). COVID-19 and your smartphone: BLE-based smart contact tracing. arXiv preprint arXiv:2005.13754. 10.1109/JSYST.2021.3055675 PMC8843047 35582390

[B61] Ngu A. H. Gutierrez M. Metsis V. Nepal S. Sheng Q. Z. (2016). IoT middleware: a survey on issues and enabling technologies. IEEE Intern. Things J. 4, 1–20. 10.1109/JIOT.2016.2615180

[B62] Noor M. M. Hassan W. H. (2019). Current research on internet of things (IoT) security: a survey. Comput. Netw. 148, 283–294. 10.1016/j.comnet.2018.11.02530513733

[B63] NRC (2020). COVID-19 Challenge: Low-Cost Sensor System for COVID-19 Patient Monitoring. NRC.

[B64] Olagnier D. Mogensen T. H. (2020). The COVID-19 pandemic in Denmark: big lessons from a small country. Cytokine Growth Fact. Rev. 53, 1–12. 10.1016/j.cytogfr.2020.05.005 32405247 PMC7217796

[B65] Opperman C. A. Hancke G. P. (2011). A generic NFC-enabled measurement system for remote monitoring and control of client-side equipment, in 2011 Third International Workshop on Near Field Communication (Hagenberg), 44–49. 10.1109/NFC.2011.11

[B66] Pacelli M. Caldani L. Paradiso R. (2006a). Textile piezoresistive sensors for biomechanical variables monitoring, in 2006 International Conference of the IEEE Engineering in Medicine and Biology Society (New York, NY), 5358–5361. 10.1109/IEMBS.2006.259287 17946696

[B67] Pacelli M. Loriga G. Taccini N. Paradiso R. (2006b). Sensing fabrics for monitoring physiological and biomechanical variables: E-textile solutions, in 2006 3rd IEEE/EMBS International Summer School on Medical Devices and Biosensors (Cambridge, MA), 1–4. 10.1109/ISSMDBS.2006.360082

[B68] Papazoglou M. P. Georgakopoulos D. (2003). Introduction: Service-oriented computing. Commun. ACM 46, 24–28. 10.1145/944217.944233

[B69] Qi J. Yang P. Waraich A. Deng Z. Zhao Y. Yang Y. (2018). Examining sensor-based physical activity recognition and monitoring for healthcare using internet of things: a systematic review. J. Biomed. Inform. 87, 138–153. 10.1016/j.jbi.2018.09.002 30267895

[B70] Ransing R. Adiukwu F. Pereira-Sanchez V. Ramalho R. Orsolini L. Teixeira A. L. S.. (2020). Mental health interventions during the COVID-19 pandemic: a conceptual framework by early career psychiatrists. Asian J. Psychiatry 51, 102085–102085. 10.1016/j.ajp.2020.102085 32413616 PMC7195073

[B71] Rechy-Ramirez E. J. Hu H. (2015). Bio-signal based control in assistive robots: a survey. Digital Commun. Netw. 1, 85–101. 10.1016/j.dcan.2015.02.004

[B72] Rogers J. (2020). Rapid: Collaborative Research: Data Analytics for Mechano-Acoustic and Physiological Monitoring of COVID19 Symptoms. National Science Foundation.

[B73] Roser M. Ritchie H. Ortiz-Ospina E. Hasell J. (2020). Coronavirus Disease (COVID-19)-Statistics and Research. Our World in data.

[B74] Sadowski S. Spachos P. Plataniotis K. N. (2020). Memoryless techniques and wireless technologies for indoor localization with the internet of things. IEEE Intern. Things J. 7, 10996–11005. 10.1109/JIOT.2020.2992651

[B75] Servick K. (2020). COVID-19 contact tracing apps are coming to a phone near you. How will we know whether they work? Science. 10.1126/science.abc9379

[B76] Shen C.-L. Huang T.-H. Hsu P.-C. Ko Y.-C. Chen F.-L. Wang W.-C.. (2017). Respiratory rate estimation by using ECG, impedance, and motion sensing in smart clothing. J. Med. Biol. Eng. 37, 826–842. 10.1007/s40846-017-0247-z 30220900 PMC6132375

[B77] Sibinski M. Jakubowska M. Sloma M. (2010). Flexible temperature sensors on fibers. Sensors 10, 7934–7946. 10.3390/s100907934 22163634 PMC3231195

[B78] Sicari S. Rizzardi A. Grieco L. A. Coen-Porisini A. (2015). Security, privacy and trust in internet of things: the road ahead. Comput. Netw. 76, 146–164. 10.1016/j.comnet.2014.11.008

[B79] Singh R. P. Javaid M. Haleem A. Vaishya R. Al S. (2020). Internet of medical things (IoMT) for orthopaedic in COVID-19 pandemic: roles, challenges, and applications. J. Clin. Orthop. Trauma. 11, 713–717. 10.1016/j.jcot.2020.05.011 32425428 PMC7227564

[B80] Sohrabi C. Alsafi Z. O'Neill N. Khan M. Kerwan A. Al-Jabir A.. (2020). World health organization declares global emergency: a review of the 2019 novel coronavirus (COVID-19). Int. J. Surg. 76, 71–76. 10.1016/j.ijsu.2020.02.034 32112977 PMC7105032

[B81] Soldatos J. Kefalakis N. Hauswirth M. Serrano M. Calbimonte J.-P. Riahi M.. (2015). Openiot: Open source internet-of-things in the cloud, in Interoperability and Open-Source Solutions for the Internet of Things, eds Podnar I. Žarko Pripužić K. Martin S. (Split; Cham: Springer International Publishing), 13–25. 10.1007/978-3-319-16546-2_3

[B82] Spachos P. Plataniotis K. (2020a). BLE beacons in the smart city: applications, challenges, and research opportunities. IEEE Intern. Things Mag. 3, 14–18. 10.1109/IOTM.0001.1900073

[B83] Spachos P. Plataniotis K. N. (2020b). BLE beacons for indoor positioning at an interactive IoT-based smart museum. IEEE Syst. J. 14, 3483–3493. 10.1109/JSYST.2020.2969088

[B84] Stavem K. Saxholm H. Smith-Erichsen N. (1997). Accuracy of infrared ear thermometry in adult patients. Intens. Care Med. 23, 100–105. 10.1007/s001340050297 9037647

[B85] Subashini S. Kavitha V. (2011). A survey on security issues in service delivery models of cloud computing. J. Netw. Comput. Appl. 34, 1–11. 10.1016/j.jnca.2010.07.006

[B86] Subbe C. P. Kinsella S. (2018). Continuous monitoring of respiratory rate in emergency admissions: evaluation of the respirasense-sensor in acute care compared to the industry standard and gold standard. Sensors 18:2700. 10.3390/s18082700 30126085 PMC6111745

[B87] Sun P. Lu X. Xu C. Sun W. Pan B. (2020). Understanding of COVID-19 based on current evidence. J. Med. Virol. 92, 548–551. 10.1002/jmv.25722 32096567 PMC7228250

[B88] Sundmaeker H. Guillemin P. Friess P. Woelfflé S. (2010). Vision and challenges for realising the internet of things, in Cluster of European Research Projects on the Internet of Things (Brussels: European Commision), 34–36.

[B89] Tan P.-N. Steinbach M. Kumar V. (2016). Introduction to Data Mining. New Delhi; London: Pearson Education India.

[B90] Tang Y.-W. Schmitz J. E. Persing D. H. Stratton C. W. (2020). Laboratory diagnosis of COVID-19: current issues and challenges. J. Clin. Microbiol. 58:e00512-20. 10.1128/JCM.00512-20 32245835 PMC7269383

[B91] Timalsina S. K. Bhusal R. Moh S. (2012). NFC and its application to mobile payment: overview and comparison, in 2012 8th International Conference on Information Science and Digital Content Technology (ICIDT2012), Vol. 1 (Jeju), 203–206.

[B92] Tobin M. J. Laghi F. Jubran A. (2020). Why COVID-19 silent hypoxemia is baffling to physicians. Am. J. Respir. Crit. Care Med. 202, 356–360. 10.1164/rccm.202006-2157CP 32539537 PMC7397783

[B93] Vandecasteele K. De Cooman T. Gu Y. Cleeren E. Claes K. Paesschen W. V.. (2017). Automated epileptic seizure detection based on wearable ECG and PPG in a hospital environment. Sensors 17:2338. 10.3390/s17102338 29027928 PMC5676949

[B94] Wang C. Horby P. W. Hayden F. G. Gao G. F. (2020). A novel coronavirus outbreak of global health concern. Lancet 395, 470–473. 10.1016/S0140-6736(20)30185-9 31986257 PMC7135038

[B95] Wang D. Hu B. Hu C. Zhu F. Liu X. Zhang J.. (2020). Clinical characteristics of 138 hospitalized patients with 2019 novel coronavirus-infected pneumonia in Wuhan, china. JAMA 323, 1061–1069. 10.1001/jama.2020.1585 32031570 PMC7042881

[B96] Wang X. Gao L. Mao S. (2017). BiLoc: Bi-modal deep learning for indoor localization with commodity 5GHZ Wifi. IEEE Access 5, 4209–4220. 10.1109/ACCESS.2017.2688362

[B97] Wang Z. Tang K. (2020). Combating COVID-19: health equity matters. Nat. Med. 26, 458–458. 10.1038/s41591-020-0823-6 32284617

[B98] Witt J. Narbonneau F. Schukar M. Krebber K. De Jonckheere J. Jeanne M.. (2011). Medical textiles with embedded fiber optic sensors for monitoring of respiratory movement. IEEE Sensors J. 12, 246–254. 10.1109/JSEN.2011.2158416

[B99] Wu Z. McGoogan J. M. (2020). Characteristics of and important lessons from the coronavirus disease 2019 (COVID-19) outbreak in China: summary of a report of 72 314 cases from the Chinese center for disease control and prevention. JAMA 323, 1239–1242. 10.1001/jama.2020.2648 32091533

[B100] Wynants L. Van Calster B. Bonten M. M. Collins G. S. Debray T. P. De Vos M.. (2020). Prediction models for diagnosis and prognosis of COVID-19 infection: systematic review and critical appraisal. BMJ 369:m1328. 10.1136/bmj.m1328 32265220 PMC7222643

[B101] Xia L. Malik A. S. Subhani A. R. (2018). A physiological signal-based method for early mental-stress detection. Biomed. Sign. Process. Control 46, 18–32. 10.1016/j.bspc.2018.06.004

[B102] Xiao L. Greenstein L. J. Mandayam N. B. Trappe W. (2009). Channel-based detection of sybil attacks in wireless networks. IEEE Trans. Inform. Forens. Secur. 4, 492–503. 10.1109/TIFS.2009.2026454

[B103] Xu W. Trappe W. Zhang Y. Wood T. (2005). The feasibility of launching and detecting jamming attacks in wireless networks, in Proceedings of the 6th ACM International Symposium on Mobile Ad Hoc Networking and Computing (Urbana-Champaign, IL), 46–57. 10.1145/1062689.1062697

[B104] Zastrow M. (2020). Coronavirus contact-tracing apps: can they slow the spread of COVID-19? Nature. 10.1038/d41586-020-01514-2 32433633

[B105] Zhai P. Ding Y. Wu X. Long J. Zhong Y. Li Y. (2020). The epidemiology, diagnosis and treatment of COVID-19. Int. J. Antimicrob. Agents 2020:105955. 10.1016/j.ijantimicag.2020.105955 PMC7138178 32234468

[B106] Zhang K. Liang X. Lu R. Shen X. (2014). Sybil attacks and their defenses in the internet of things. IEEE Intern. Things J. 1, 372–383. 10.1109/JIOT.2014.2344013

[B107] Zhang Z.-K. Cho M. C. Y. Wang C.-W. Hsu C.-W. Chen C.-K. Shieh S. (2014). IoT security: ongoing challenges and research opportunities, in 2014 IEEE 7th International Conference on Service-Oriented Computing and Applications, (Matsue:IEEE). 230–234. 10.1109/SOCA.2014.58

[B108] Zhen R. Jin Y. Hu Q. Shao Z. Nikitakos N. (2017). Maritime anomaly detection within coastal waters based on vessel trajectory clustering and naïve bayes classifier. J. Navigat. 70:648. 10.1017/S0373463316000850

[B109] Zheng Y.-Y. Ma Y.-T. Zhang J.-Y. Xie X. (2020). COVID-19 and the cardiovascular system. Nat. Rev. Cardiol. 17, 259–260. 10.1038/s41569-020-0360-5 32139904 PMC7095524

